# Private Repertories

**Published:** 1891-03

**Authors:** W. A. Yingling

**Affiliations:** Nonchalanta, Kansas


### PRIVATE REPERTORIES.

W. A. Yingling, M. D., Nonchalanta, Kansas.

The extensive materia medica of the homœopathic system of medicine is beyond the capacity of the most retentive and trusty memory. The richness of the drug symptoms, and the necessity for the consideration of every and all indications of each remedy in order to obtain the absolute curative, adds materially to the necessary matter to be considered in each case. As a flash prescription is hazardous to the life of the patient, as well as to the honor and integrity of the physician, some means by which this extensive material may be employed becomes very important, and, I may say, very necessary. As no mind can retain it all, and as searching the *Materia Medica* till the remedy is ascertained is to waste too much important time, some means must be had by which the material may be at command. This agency is the repertory, or index of the fullest symptom list to be obtained. From the necessity of the case every repertory must be more or less deficient, and in a short time become inadequate to sweep the whole horizon of the *Materia Medica* sky, as each week, almost, adds new material, and even new indications of the oldest remedies. Homœopathy is progressive, not in changing the *only* law of cure, but in adding material for the complete demonstration of that law. One important consideration is that, so opposite to the allopathic school, the old material never becomes obsolete with the acquisition of the new, but the new and old become incorporated into the one great system of curative medicine.

From these considerations we see the importance of each physician having his own private repertory in which he may record the progress of his school, and be ready to give his patients the best possible return for their money, and at the same time honor his profession by integrity of purpose, and proficiency in the healing art. We urge the private repertory from three considerations. Without his own private repertory the physician loses many of the best indications of remedies obtained from his own experience, and that of others, as recorded in the various medical journals. Again, without it he loses the benefit of his journalistic and other readings, for the mind will not always recall the remedy when needed, which would be at hand if properly recorded in his own index. It is hardly necessary to mention the waste of time frequently occasioned by searching the books to find something that one remembers fully well to have read, but can't tell just where to find it.

Each mind grasps ideas differently, or rather records ideas with different symbols. Thus each repertory has the bias of the mental peculiarities of its author. This is manifest to each one when he remembers the time consumed in searching the repertories for some symptom that he knew must be recorded, but could not find the indicating word to enable him to turn to it readily. It is simple, and easily comprehended when found; he wonders why he did not think of it, but the press of other thoughts or time prevented. His private repertory would have saved him valuable time.

In keeping a repertory the word or words of the symptom must be recorded with which the person is accustomed to think of the idea. Persons differ in this regard. One may always use and think of the word "womb," another of the "uterus," and still another may turn to "the female organs." If the record is made in the person's own thought-word, much time will be saved. This applies to all symptoms, organs, etc., as recorded in this private repertory. To facilitate the finding of what we wish there should be as many entries as there are ideas, organs, or leading words in the symptom sentence. If the sentence does not contain the word with which the recorder is most familiar, he should make the record under the word or words which come to his mind most naturally, or a reference from them. To illustrate what I mean I will give a little of my own experience, though modesty would prevent. I received a call for medicine by mail, in which the symptom, "Sensation of a *string* around the body" was prominent, and mentioned several times. I was in a hurry, busy, and pressed for time. I thought of "string," but of no other word of the same idea. I searched the repertories without success. I knew the symptom was there. I worked, and thought hard, losing much valuable time, but from some mental state I failed to look in the right place till I asked myself what other word would imply the same idea. When "hoop," "band," came to mind I had no further trouble. I at once made a record in this way: "String, see Hoop or Band." One minute's time in recording the word would have saved me a full hour, and much chagrin.

The source of the material of the private repertory must come from personal experience, the *Materia Medica*, and references to other repertories, owned by the individual. I make it a rule to record everything that has the appearance of being in any way likely to be needed, and even record symptoms of the reliability of which I am uncertain, but in such a case I record the doubt also by the question mark, thus (?), which is afterward erased, if found to be reliable. By this means all the experience recorded in my journals is preserved, and ready at hand for speedy reference, and the new remedies, as given in the journals, can be as readily used as the older ones, for I have a full repertory of them, made by my own hands.

The repertories don't usually give the symptoms in full, but merely refer to the remedies; hence in reading the *Materia Medica* it is well to record any peculiar, odd, or characteristic symptom, or sensation, so as to be at once referred to without looking at half a dozen remedies to find it. It is also a saving of time to contra-distinguish remedies having symptoms very similiar, or the same symptom with a shade of difference, by giving the peculiar symptoms of each under the proper headings, and at the same place.

The mere fact of recording the symptoms impresses them on the mind, and they are more likely to be remembered by the plan. Another benefit which should cause every one to have and use a private repertory is that the necessity of *weighing* and considering symptoms preparatory to recording them gives the mind the power of discrimination, which is so very important in a successful prescription. Carefully keeping a private repertory, where one tries to make it the best and most useful to himself, causes the mind to be on the alert for material, and thus saves all that is useful and beneficial. Always read the medical journals, with pencil and paper in hand to note down anything worth recording, and as it is to be recorded, and during the spare moments, or by a trusted member of the family, the notes may be properly recorded. In the busy season a good many notes may be accumulated, but being written as they are to be recorded, no trouble will be found when time permits to permanently secure them in the proper places. It is very judicious to have a few slips of blank paper in the *Materia Medica* for the purpose of noting any symptom which may impress itself on the mind, when studying or searching for something else, as sufficiently important to require recording. It will be found that almost every time the *Materia Medica* is picked up some note will be made, as the importance of symptoms is brought out by the necessities of clinical experience. The wise man fortifies himself in the time of ease for the hour of trial and necessity.

But my article is becoming too lengthy. I will add a few lines as to the way to keep a private repertory, or rather the way *I* do, hoping some one may be benefited. Secure a book, well bound and of good paper, of about 600 pages, with a margin of one inch at the top and at the left-hand side of each page; lines one-quarter inch apart, and paged with small figures. As the book is for constant use, I had one made to order of the best thin linen-paper, and bound in flexible leather so as to open readily in a smooth page. The next step is to index it by using the Index Rerum letters, thus: Aa, Ae, Ai, Ao, Au, Ba, Be, Bi, Bo, Bu, Ca, Ce, Ci, Co, Cu, etc., with *each* letter of the alphabet, giving so many pages to each combination. This represents the first *letter* and the first *vowel* of each word that is the key of the sentence. If I desired to record the symptom, "profuse, scaly dandruff on the scalp, Sanicula,' I would select "Scalp," and under "Sa," the first letter and first vowel of the word, I would write: "Scalp, profuse, scaly dandruff on, Sanicula." The word "scalp," being the key of the symptom (so far as recording is concerned), should be written in the *side* margin mentioned. The "Sa" and all others being printed in plain letters by the pen in the *top* margin. By this means the repertory is as easily referred to as the dictionary, and in the same way. Care must be taken to allow sufficient space between each separate section. I allow a half page to "scalp," two pages to "Urine," "Cough," one to "Abdomen," one-fourth to "Knee," "Hand," "Finger," "Toe," etc., each, of course, in its respective place.

When a section, for instance, all of "Ae," is full of recorded matter, refer to the page to which the "Ae" is carried forward by writing at the bottom of the *last* page containing "Ae," the number of the new page, thus, "☞ Forward, page 420," and at the top of the new page write, "From page 10." Where a new subject is entered on the continued page, no indication to the former page need be made, but where a subject first entered on the original page is continued on the added page, it saves trouble to write in the margin of the first entry the number of the new page, simply, —420—, and in the margin of the second and continued entry the old page. The necessity of this will be apparent when we come to look over the first entry, which is always first done, and find it full. Instead of looking to ascertain whether it is continued, and to find the page, the figures in the margin tell me at once whether continued and the page. The marginal figures of the continued entry refer me at once to the original entry. This makes a continual connection between different entries of the same subject, and one most easy to understand.

Ordinary symptoms are placed under the name of the disease, while special symptoms are placed under the name of the part of the body, or under Aversion, Desire, Aggravation, Amelioration, etc., as the case may be, unless especially important, when the entry should be made under the special name. To place everything a sick man might desire under "Desire" would make that section too full, but by placing "Desire for beer" under "Beer" would facilitate space and search. Every one must use his own judgment in this matter. I give my own mode, which I find to be handy.

All references to journals can be made by abbreviations, so as to save space, and at the same time be legible. I transfer a reference from my own repertory in illustration:

"Skin diseases. H. P. 8–480, 10–74." This means that in THE HOMŒOPATHIC PHYSICIAN, vol. 8, page 480, and vol. 10, page 74, will be found something of interest on skin diseases. "Sight. M. A. 22–248." In the *Medical Advance*, vol. 22, page 248, is an article on sight.

It is well to refer to all articles in the journals of a general character, like "The Dose," "The Potency," "Hahnemann," etc., so as to be able to refer to them immediately without the necessity of search. I keep an index to all subjects that refer in any way to my profession.

Refer to all notices of remedies in the journals, beside recording the indications; then, when one desires to study a remedy, he will have at his command all journal notices of said remedy. This is simply done. "Agaricus-musc. H. P. 10–144." "Saccharum-lac. H. P. 10–137." "Ailanthus-glan. H. P. 7–456. H. P. 8–67, 218." In all clinical cases refer to the remedy and the disease also. This plan gives us the cures effected by the remedy, and the remedy used in the disease, both of which are important.

I have but one excuse for writing this article. I would have been thankful for one like it some years ago. There may be others now who will be benefited by this one, imperfect as it is. "It is more blessed to give than to receive." Let others who have a better plan give it to the profession.

[We have used an index, as suggested by Dr. Yingling in the foregoing article, for years, and find it extremely valuable. We were not obliged, however, to make our own index. There are such indexes, patented, in the market, with thumb spaces showing the letters in the margin, enabling one to turn to the place desired immediately. We cannot too strongly urge upon our readers the need of following Dr. Yingling's advice in this matter. He has evidently given the subject careful attention, as the scope of his paper sufficiently attests. W. M. J.]

